# Dynamics of adaptive immunity against phage in bacterial populations

**DOI:** 10.1371/journal.pcbi.1005486

**Published:** 2017-04-17

**Authors:** Serena Bradde, Marija Vucelja, Tiberiu Teşileanu, Vijay Balasubramanian

**Affiliations:** 1 Initiative for the Theoretical Sciences, The Graduate Center, CUNY, New York, New York, United States of America; 2 David Rittenhouse Laboratories, University of Pennsylvania, Philadelphia, Pennsylvania, United States of America; 3 Center for Studies in Physics and Biology, The Rockefeller University, New York, New York, United States of America; 4 Department of Physics, University of Virginia, Charlottesville, Virginia, United States of America; University of California Irvine, UNITED STATES

## Abstract

The CRISPR (clustered regularly interspaced short palindromic repeats) mechanism allows bacteria to adaptively defend against phages by acquiring short genomic sequences (spacers) that target specific sequences in the viral genome. We propose a population dynamical model where immunity can be both acquired and lost. The model predicts regimes where bacterial and phage populations can co-exist, others where the populations exhibit damped oscillations, and still others where one population is driven to extinction. Our model considers two key parameters: (1) ease of acquisition and (2) spacer effectiveness in conferring immunity. Analytical calculations and numerical simulations show that if spacers differ mainly in ease of acquisition, or if the probability of acquiring them is sufficiently high, bacteria develop a diverse population of spacers. On the other hand, if spacers differ mainly in their effectiveness, their final distribution will be highly peaked, akin to a “winner-take-all” scenario, leading to a specialized spacer distribution. Bacteria can interpolate between these limiting behaviors by actively tuning their overall acquisition probability.

## Author summary

The CRISPR system in bacteria and archaea provides adaptive immunity by incorporating foreign DNA (spacers) into the genome, and later targeting DNA sequences that match these spacers. The way in which bacteria choose spacer sequences from a clonal phage population is not understood. Our model considers competing effects of ease of acquisition and effectiveness against infections in shaping the spacer distribution. The model suggests that a diverse spacer population results when the acquisition rate is high, or when spacers are similarly effective. At moderate acquisition rates, the spacer distribution becomes highly sensitive to spacer effectiveness. There is a rich landscape of behaviors including bacteria-phage coexistence and oscillations in the populations.

## Introduction

**Fig 1:**
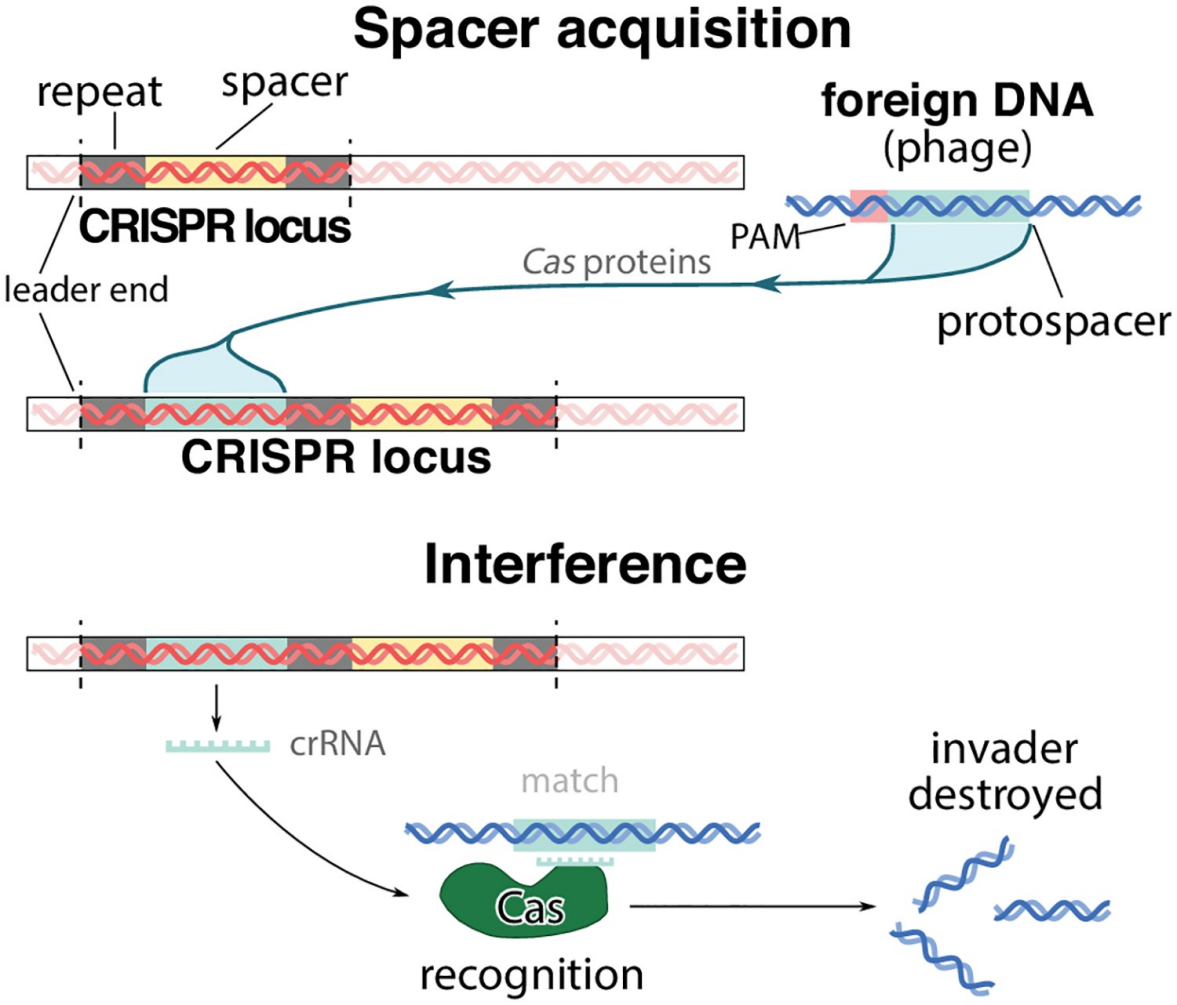
Schematic of the CRISPR acquisition and interference mechanism. PAM stands for protospacer adjacent motif, a short sequence necessary for protospacer recognition by the *Cas* proteins.

Bacteria and archaea can combat viral infections using innate mechanisms (*e.g.*, abortive infection, surface exclusion and restriction modification systems) that are not specific to particular threats [1–3]. Some species also exhibit an adaptive immune system based on CRISPR (clustered regularly interspaced short palindromic repeats) interference, which allows bacteria to specifically target and cleave exogenous genetic material from previously encountered phages and other genetic elements [4–7]. The system works by incorporating short (30–70 bp) sequences, dubbed “spacers”, into the bacterial genome, in between repeated CRISPR elements (Fig 1). The spacers are acquired from the “protospacer” regions in the genome of infecting phage. CRISPR Type I and II require the presence of a “protospacer adjacent motif” (PAM) upstream of a protospacer for recognition by the CRISPR proteins [8]. The PAM sequence is thought to play a role in the avoidance of auto-immune targeting [9]. While the PAM and the first few nucleotides of the protospacer (the “seed” region) need to match almost perfectly for CRISPR interference [6], there is significant tolerance to mutations in the rest of the spacer [10].

Over the whole viral genome, there can be tens or hundreds of protospacers, and the way in which the CRISPR acquisition mechanism selects between these is not fully understood [11]. Experiments show that after several hours of exposure of bacteria to phage, different spacers occur with different frequencies, with a handful being much more abundant [12]. Importantly, many of the highly abundant spacers recur during repetition of the experiments, suggesting that their over-representation is not simply the result of amplification of spacers that are randomly acquired at the early stage of infection. There are three main possible sources of selective pressure on spacers. One is a bias in acquisition that may arise either when some protospacers are easier to acquire by the CRISPR proteins than others [13], or when some protospacers are more conserved in the viral population, and thus more abundant and more likely to be acquired. Another possible source of selective pressure is that some spacers might be more effective than others at clearing viral infections and so provide a selective advantage for the host [4, 10]. Finally, the acquisition of some spacers might be “primed” by the presence of other spacers in the CRISPR locus [6, 11, 14, 15].

We construct a population dynamical model for bacteria that use CRISPR-based immunity to defend against phage. Our model predicts that even when dilution is negligible, wild-type and spacer-enhanced bacteria can co-exist with phage, provided there is spacer loss. Previous Lotka-Volterra-like ecological models have demonstrated a mechanism for coexistence between three species with bounded populations, but, unlike the scenario we describe, they required dilution and significant differences in the growth rates of the two prey species [16]. To understand the factors that affect spacer diversity, we compare two scenarios: (a) different spacers are acquired at different rates; (b) different spacers provide different advantages, *e.g.*, in growth rate or survival rate, to the host. We derive analytical results for the spacer distribution that is reached at late times, and show that the spacer-effectiveness model favors a peaked distribution of spacers while the spacer-acquisition model favors a more diverse distribution. Higher rates of spacer acquisition also lead to higher diversity. We expect that greater spacer diversity will be important for defending against a mutating phage landscape, while a peaked spacer distribution will confer stronger immunity against a specific threat. Our model predicts that bacteria can negotiate this tradeoff by controlling the overall rate at which spacers are acquired, *i.e.*, by modifying the expression of the *Cas* proteins, necessary for acquisition [6].

## Model

We consider bacteria that start with a CRISPR cassette containing no spacers, a scenario that has been proven functional *in vivo* [17]. We focus on the early dynamics of the bacterial population after being infected with phage in which each bacterial cell acquires at most one spacer. Experiments suggest that this scenario may be appropriate for bacteria-phage interactions lasting about a day, which allows most of the bacterial population to become immune to the infecting phage, but is not enough time for viral escapers that can avoid the bacterial defenses to become abundant [12, 18]. In the absence of escapers, the acquisition of new spacers against the same virus is slow [14], extending the duration for which our single spacer approximation is valid. As time goes by, the virus will mutate and the bacteria need to acquire new spacers to keep up with the mutants; we leave the study of this co-evolution to future work, and focus here on the early dynamics of spacer acquisition.

Even if each bacterial cell only has time to acquire at most one spacer, the population as a whole will contain a diverse spacer repertoire [12, 19, 20]. Here we propose a model of bacteria-phage dynamics to understand the distribution of spacers in the population. As a warmup, we first study the case where the virus contains only a single protospacer, then we generalize the model to the case of many protospacers where acquisition probability and effectiveness can depend on the type.

### One spacer type

**Fig 2:**
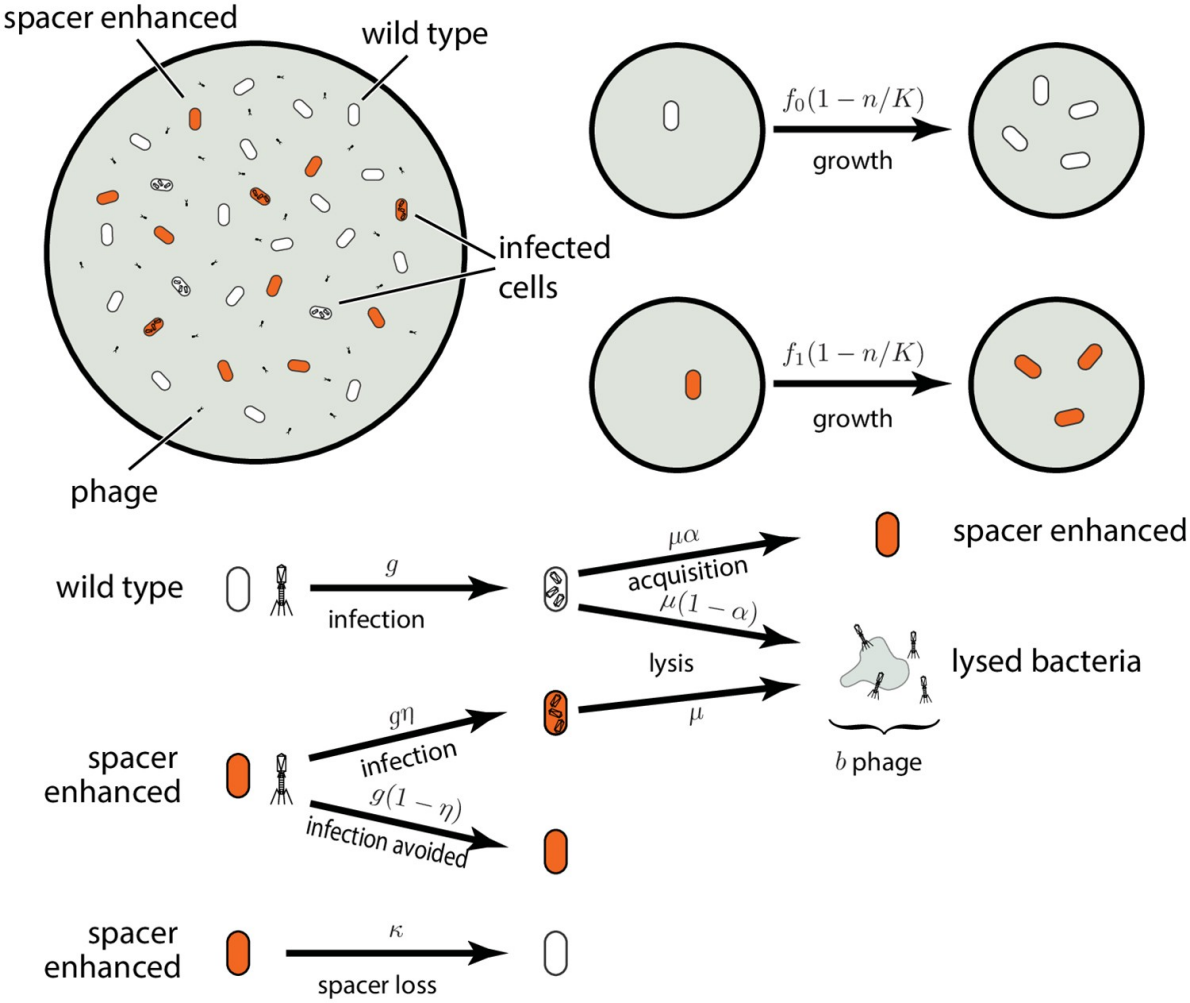
Model of bacteria and phage dynamics. Bacteria are either wild type or spacer enhanced, grow at different rates *f*_0_ and *f*_1_ and can be infected by phage with rates *g* and *ηg*. Spacers can be acquired during infection with a probability *α* and spacers are lost at a rate *κ*.

To set the stage, we will first introduce the dynamics of a model where viruses present a single protospacer. In this case, all immune bacteria have the same spacer. We will assume logistic growth of the bacteria [21]. The relevant processes are sketched in Fig 2 and, assuming a well-mixed population, can be translated into a set of ordinary differential equations:

$$\begin{array}{ll} {\dot{n}}_{0} & =f_{0}\left(1-\frac{n}{K}\right)n_{0}+\kappa n_{1}-gvn_{0},\\ {\dot{n}}_{1} & =f_{1}\left(1-\frac{n}{K}\right)n_{1}-\kappa n_{1}-\eta gvn_{1}+\alpha \mu I_{0},\\ {\dot{I}}_{0} & =gvn_{0}-\mu I_{0},\\ {\dot{I}}_{1} & =\eta gvn_{1}-\mu I_{1},\\ \dot{v} & =b\left(1-\alpha \right)\mu I_{0}+b\mu I_{1}-gv\left(n_{0}+n_{1}\right). \end{array}$$ (1)

Here the dot represents the derivative with respect to time, *n*_0_ is the number of “wild type” bacteria that do not contain any spacers, *n*_1_ is the number of “spacer enhanced” bacteria that have acquired the spacer, *I*_0_ is the number of wild-type infected bacteria, and *I*_1_ is the number of spacer enhanced but infected bacteria (which is possible because spacers do not provide perfect immunity). The sizes of the bacterial and phage populations are

$$n=n_{0}+n_{1}+I_{0}+I_{1}$$

and *v* respectively.

The first term in the first two equations in (Eq 1) describes logistic growth of the bacteria with maximum growth rates *f*_*i*_ and a carrying capacity *K*. These equations allow for the possibility that spacer enhanced bacteria may grow at a different rate than the wild type because of possible spacer toxicity due to auto-immune interactions or due to increased metabolic rate arising from expression of CRISPR (*Cas*) proteins and/or CRISPR RNA (crRNA). However, there is evidence [12, 22] that these growth rate differences are small so that *r* = *f*_1_/*f*_0_ ≈ 1. We also assume that spacers can be lost at a rate *κ* (second term in the first and second equations) allowing bacteria to revert to wild type [22–24]. Bacteria become infected with different rates depending on their type—wild type are always infected if they encounter phage, but spacer enhanced bacteria may evade infection. Taking *g* to be the encounter rate, wild type are infected at a rate *g* while spacer enhanced bacteria are infected at a rate *ηg* where *η* < 1 (third terms of the first and second equations). We can think of *η* as a “failure probability” of the spacer as a defense mechanism, or alternatively, of 1 − *η* as a measure of the “effectiveness” of the spacer against infections. Finally, some infected wild-type bacteria survive and acquire a spacer with probability *α* (last term in the second equation). We can imagine that this acquisition occurs in the course of an infection that is unsuccessful because the phage is ineffective or because of innate immune mechanisms, while nevertheless allowing the bacterial cell access to genetic material of the phage. We are neglecting the possibility that spacers might also be acquired via horizontal gene transfer without an infection.

The dynamics of the infected bacteria is given in the third and fourth equations in (Eq 1). We assume that infected bacteria do not divide. So the number of infected bacteria grows only because of new infections (first terms in the equations), and declines due to lysis or successful defense followed by acquisition of spacers (second term). The lysis rate *μ* depends on properties of the phage including the burst factor *b* (*i.e.*, the number of viral particles produced before lysis). More specifically, there is a delay between infection and lysis because it takes some time for the virus to reproduce. We are approximating this delay with a stochastic process following an exponential distribution with timescale 1/*μ* [25, 26].

Finally, the last equation describes the dynamics of free phage. The first two terms model viral replication. Phage that duplicate in infected bacteria produce *b* new copies after cell lysis. The first term describes this process in infected wild type bacteria that do not acquire a spacer and become immune. The second term describes the lysis of bacteria that were infected despite having a spacer. We could imagine that a small number of spacer enhanced bacteria that become infected then become resistant again, perhaps by acquiring a second spacer. We neglect this because the effect is small for two reasons—acquisition is rare, *α* ≪ 1, and because we assume that the spacer is effective, *η* ≪ 1, such that *I*_1_ is small. The approximation *η* ≪ 1 is supported by experimental evidence that shows that a single spacer seems often sufficient to provide almost perfect immunity [4].

For simplicity, our model does not include the effects of natural decay of phage and bacteria as these happen on timescales that are relatively long compared to the dynamics that we are studying. Likewise, we did not consider the effects of dilution which can happen either in controlled experimental settings like chemostats, or in some kinds of open environments. In S1 File we show that dilution and natural decay of typical magnitudes do not affect the qualitative character of our results.

We can also write an equation for the total number of bacteria *n*:

$$\dot{n}=f_{0}\left(n_{0}+rn_{1}\right)\left(1-\frac{n}{K}\right)-\mu \left(1-\alpha \right)I_{0}-\mu I_{1}\;,$$ (2)

where we used the notation *r* = *f*_1_/*f*_0_. The total number of bacteria is a useful quantity, since optical density measurements can assess it in real time.

### Multiple spacer types

Typically the genome of a given bacteriophage contains several protospacers as indicated by the occurrence of multiple PAMs. Even though in the short term each bacterial cell can acquire only one spacer type, at the level of the whole population many types of spacers will be acquired, corresponding to the different viral protospacers. Experiments show that the frequencies with which different spacers occur in the population are highly non-uniform, with a few spacer types dominating [12]. This could happen either because some spacers are easier to acquire than others, or because they are more effective at defending against the phage.

We can generalize the population dynamics in (Eq 1) to the more general case of *N* spacer types. Following experimental evidence [22] we assume that all bacteria, with or without spacers, grow at similar rates (*f*)—the effect of having different growth rates is analyzed in S1 File. We take spacer *i* to have acquisition probability *α*_*i*_ and failure probability *η*_*i*_. As before, we can alternatively think of 1 − *η*_*i*_ as the effectiveness of the spacer against infection. The dynamical equations describing the bacterial and viral populations become

$$\begin{array}{ll} {\dot{n}}_{0} & =f\left(1-\frac{n}{K}\right)n_{0}+\kappa \sum_{i=1}^{N}n_{i}-gvn_{0},\\ {\dot{n}}_{i} & =f\left(1-\frac{n}{K}\right)n_{i}-\kappa n_{i}-\eta_{i}gvn_{i}+\alpha_{i}\mu I_{0},\\ {\dot{I}}_{0} & =gvn_{0}-\mu I_{0},\\ {\dot{I}}_{i} & =\eta_{i}gvn_{i}-\mu I_{i},\\ \dot{v} & =b\left(1-\sum_{i=1}^{N}\alpha_{i}\right)\mu I_{0}+b\mu \sum_{i=1}^{N}I_{i}-gv\left(n_{0}+\sum_{i=1}^{N}n_{i}\right) \end{array}$$ (3)

where *n*_*i*_ and *I*_*i*_ are the numbers of healthy and infected bacteria with spacer type *i*, and $\alpha =\sum_{i=1}^{N}\alpha_{i}$ is the overall probability of wild type bacteria surviving and acquiring a spacer, since the *α*_*i*_ are the probabilities of disjoint events. This implies that *α* < 1. The total number of bacteria is governed by the equation

$$\dot{n}=f\left(1-\frac{n}{K}\right)\left(n-\sum_{i=0}^{N}I_{i}\right)-\mu \left(1-\alpha \right)I_{0}-\mu \sum_{i=1}^{N}I_{i}\;.$$ (4)

## Results

The two models presented in the previous section can be studied numerically and analytically. We use the single spacer type model to find conditions under which host–virus coexistence is possible. Such coexistence has been observed in experiments [18] but has only been explained through the introduction of as yet unobserved infection associated enzymes that affect spacer enhanced bacteria [18]. Host-virus coexistence has been shown to occur in classic models with serial dilution [16], where a fraction of the bacterial and viral population is periodically removed from the system. Here we show additionally that coexistence is possible without dilution provided bacteria can lose immunity against the virus. We then generalize our results to the case of many protospacers where we characterize the relative effects of the ease of acquisition and effectiveness on spacer diversity in the bacterial population.

### Extinction versus coexistence with one type of spacer

**Fig 3:**
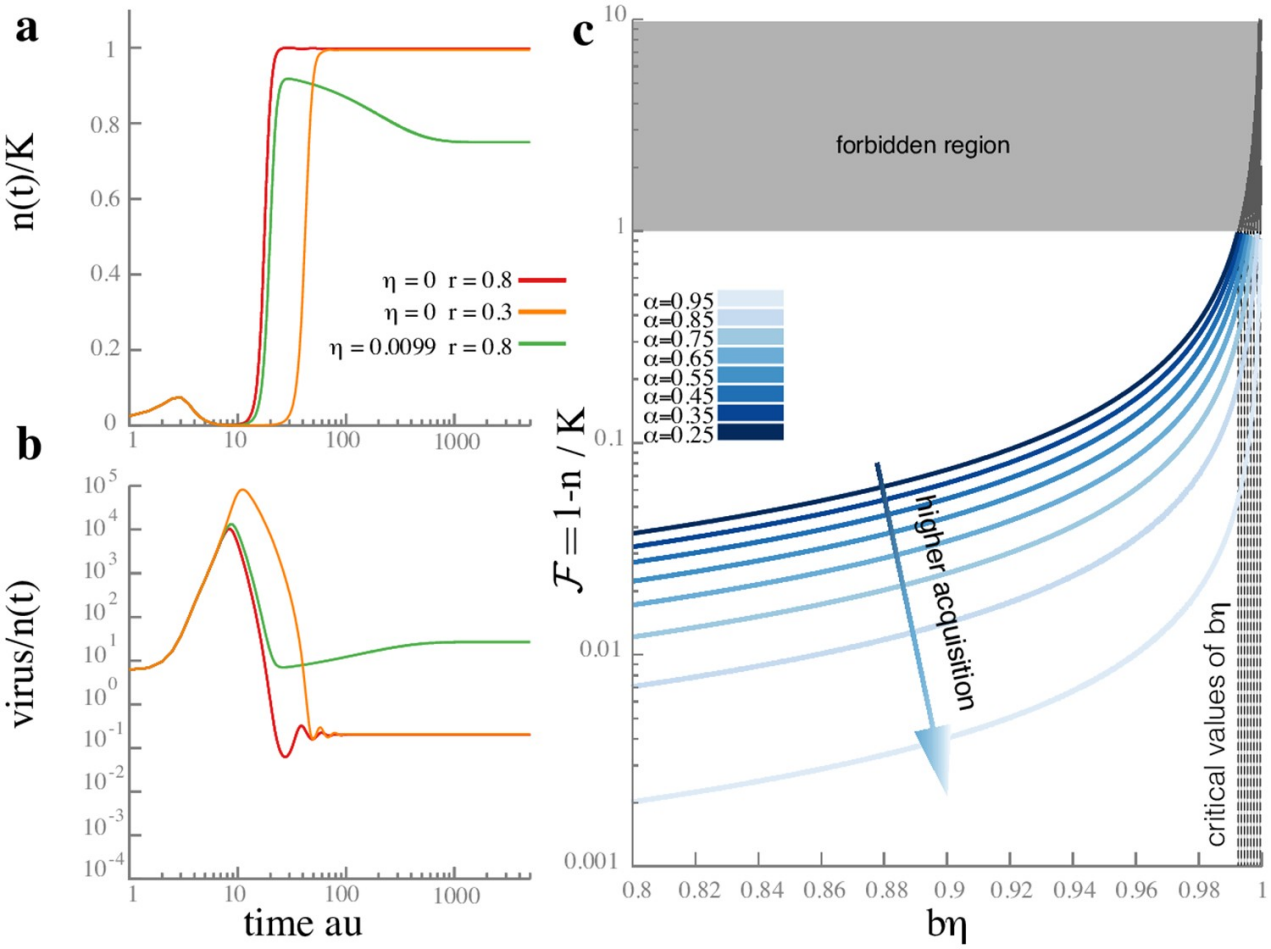
Model of bacteria with a single spacer in the presence of lytic phage. (Panel **a**) shows the dynamics of the bacterial concentration in units of the carrying capacity *K* = 10^5^ and (Panel **b**) shows the dynamics of the phage population. In both panels, time is shown in units of the inverse growth rate of wild type bacteria (1/*f*_0_) on a logarithmic scale. Parameters are chosen to illustrate the coexistence phase and damped oscillations in the viral population: the acquisition probability is *α* = 10^−4^, the burst size upon lysis is *b* = 100. All rates are measured in units of the wild type growth rate *f*_0_: the adsorption rate is *g*/*f*_0_ = 10^−5^, the lysis rate of infected bacteria is *μ*/*f*_0_ = 1, and the spacer loss rate is *κ*/*f*_0_ = 2 × 10^−3^. The spacer failure probability (*η*) and growth rate ratio *r* = *f*_1_/*f*_0_ are as shown in the legend. The initial bacterial population was all wild type, with a size *n*(0) = 1000, while the initial viral population was *v*(0) = 10000. The bacterial population has a bottleneck after lysis of the bacteria infected by the initial injection of phage, and then recovers due to CRISPR immunity. Accordingly, the viral population reaches a peak when the first bacteria burst, and drops after immunity is acquired. A higher failure probability *η* allows a higher steady state phage population, but oscillations can arise because bacteria can lose spacers (see also S1 File). (Panel **c**) shows the fraction of unused capacity at steady state (Eq 6) as a function of the product of failure probability and burst size (*ηb*) for a variety of acquisition probabilities (*α*). In the plots, the burst size upon lysis is *b* = 100, the growth rate ratio is *f*_1_/*f*_0_ = 1, and the spacer loss rate is *κ*/*f*_0_ = 10^−2^. We see that the fraction of unused capacity diverges as the failure probability approaches the critical value *η*_*c*_ ≈ 1/*b* (Eq 7) where CRISPR immunity becomes ineffective. The fraction of unused capacity decreases linearly with the acquisition probability following (Eq 6).

The numerical solution of the single-spacer population dynamics model is shown in Fig 3a and 3b for different parameter choices; more details can be found in S1 File. In all cases, the bacterial population grows initially because infected bacteria do not die instantly. If the viral load is high, most bacteria are quickly infected and growth starts slowing down since infected bacteria cannot duplicate. After a lag of order 1/*μ*, where *μ* is the rate at which infected bacteria die, the population declines due to lysis. If the viral load is low, the division of healthy bacteria dominates the death of infected ones, until the viral population released by lysis becomes large enough to infect a substantial fraction of the bacteria.

Some infected bacteria acquire the spacer that confers partial immunity from the phage. During every encounter between a bacterial cell and a virus, there is a probability *η* that the spacer will be ineffective. Thus the expected increase in the number of viral particles following an encounter is *bη* − 1 where *b* is the viral burst size following lysis of an infected cell. If *η* > 1/*b*, the viral growth cannot be stopped by CRISPR immunity and the bacteria are eventually overwhelmed by the infection. Thus whenever the virus has a high burst factor, only a population with an almost perfect spacer (the failure probability *η* < 1/*b* ≪ 1) is able to survive infection.

The viral concentration has a more complex dynamics—it typically reaches a maximum, then falls due to CRISPR interference, and starts oscillating at a lower value (Fig 3b). The initial rise of the viral population occurs because of successful infections of the wild-type bacteria. But then, the bacteria which have acquired effective spacers grow exponentially fast, virtually unaffected by the presence of the virus. Since the virus is adsorbed by immune bacteria, but are cleaved by CRISPR and cannot duplicate, the viral population declines exponentially. However, as the population of spacer-enhanced bacteria rises, so does the population of wild type, because of the constant rate of spacer loss. This starts a new growth period for the virus, leading to the oscillations seen in simulations. When spacer effectiveness is low, the virus can still have some success infecting spacer-enhanced bacteria, and the oscillations are damped. It would be interesting to test whether large oscillations in the viral concentration can be seen in experiments to see if these are compatible with measured estimates of the rate of spacer loss *κ* in the context of our model [22, 27].

Varying the growth rate of the bacteria with CRISPR relative to the wild type has a strong effect on the length of the initial lysis phase and the delay before exponential decay of the viral population sets in. In contrast, a lower effectiveness of the CRISPR spacer (*i.e.*, larger failure probability *η*; green line in Fig 3b) leads to a higher minimum value for the viral population and weaker oscillations. This could potentially be used to disentangle the effects of growth rate and CRISPR interference on the dynamics.

After a transient period, the dynamics will settle into a stationary state. The transient is shorter if the spacer enhanced growth rate *f*_1_ is high, or if the failure probability of the spacer *η* is low (Fig 3, panel a and b). Depending on the choice of initial values and the parameters, there are different steady states. If spacers are never lost (*κ* = 0), we found numerically that a stable solution occurs when viruses go extinct and infections cease (*v* = 0, *I*_0,1_ = 0). In this case, the total number of bacteria becomes stationary by reaching capacity (*n* = *K*), which can only happen when the spacer is sufficiently effective (*η* < 1/*b*). Otherwise bacteria go extinct first (*n* = 0) and then the virus persists stably.

A more interesting scenario occurs when spacers can be lost (*κ* ≠ 0). In this case coexistence of bacteria and virus (*n* > 0 and *v* > 0) becomes possible (see SI for an analytic derivation). In this case, the bacteria cannot reach full capacity at steady state—we write n=K(1-F), where the factor

$$F=1-\frac{n}{K}$$ (5)

represents the fraction of unused capacity. The general expression for F is given in the SI, and simplifies when the wild type and spacer enhanced bacteria have the same growth rate (*f*_1_ = *f*_0_) to

$$F=\frac{\kappa }{f_{0}}\frac{b(1-\alpha )-1}{\left(b-1)(1-b\eta \right)}\;.$$ (6)

Fig 3c shows the dependence of F on the failure probability of the spacer (*η*) multiplied by the burst factor (*b*). We see that even if the spacer is perfect (*η* = 0) the steady state bacterial population is less than capacity (F>0). These equations are valid when F<1—this is only possible if the spacer failure probability (*η*) is smaller than a critical value (*η*_*c*_), where

$$\eta_{c}=\frac{1}{b}\left(1-\frac{\kappa }{f_{0}}\frac{b(1-\alpha )-1}{b-1}\right)+O\left(\frac{r-1}{b^{2}}\right)\;,$$ (7)

where as before *r* = *f*_1_/*f*_0_. This coexistence phase has been found in experiments [18] where the bacterial population reaches a maximum that is “phage” limited like in our model.

In the coexistence phase, the wild type persists at steady state, as observed in experiments [18]. In our model, the ratio of spacer-enhanced and wild-type bacteria is

$$\frac{n_{1}}{n_{0}}=\frac{b(1-\alpha )-1}{1-b\eta }\;.$$ (8)

This ratio does not depend on the growth rates of the two types of bacteria (*f*_1_ *vs.* *f*_0_). So, given knowledge of the burst size *b* upon lysis, the population ratio in (Eq 8) gives a constraint relating the spacer acquisition probability *α* and the spacer failure probability *η*. Thus, in an experiment where phage are introduced in a well mixed population of wild type and spacer enhanced bacteria, (Eq 8) presents a way of measuring the effectiveness of a spacer, provided the machinery for acquisition of additional spacers is disabled (*α* = 0) (*e.g.*, by removing specific *Cas* proteins) [4, 28]. Plugging the effectiveness values measured in this way into our model could then be used to predict the outcome of viral infections in bacterial colonies where individuals have different spacers, or have the possibility of acquiring CRISPR immunity.

The lysis timescale 1/*μ* for infected cells affects the duration of the transient behavior of the population, as described above. The longer this timescale, the longer it takes to reach the steady state. However, the actual size of the steady state population is not dependent on *μ* because this parameter controls how long an infected cell persists, but not how likely it is to survive. This is analyzed in more detail in S1 File.

In previous models, coexistence of bacteria and phage was achieved by hypothesizing the existence of a product of phage replication that specifically affects spacer-enhanced bacteria compared to wild type [18]. Here we showed that coexistence is obtained more simply if bacteria can lose spacers, a phenomenon that has been observed experimentally [22, 23]. More specifically, in our model coexistence requires two conditions: (1) spacer loss (*κ* > 0), and (2) the failure probability of spacers is smaller than a critical value (*η* < *η*_*c*_). Our model also reproduces an effect observed by [18], namely that the steady state bacterial population is reduced by the presence of virus. While this may seem intuitive, previous population dynamics models have not reproduced this finding, which depends critically in our model on the rate of spacer loss.

### Effectiveness versus acquisition from multiple spacers

We can now proceed to analyze the case where multiple protospacers are presented. As before, when we analyze the multiple spacer model, the most interesting case is when the virus and bacteria can co-exist. The bacteria do not generally fill their capacity when this happens. The fraction of unused capacity (F=1-n/K) can be characterized using the average failure probability ($\bar{\eta }$):

$$\begin{array}{cc} F & =\frac{\kappa }{f}\frac{b(1-\alpha )-1}{\left(1-b\bar{\eta }\right)\left(b-1\right)}\;,\\ \bar{\eta } & =\frac{\sum_{i=1}^{N}\eta_{i}n_{i}}{\sum_{i=1}^{N}n_{i}}\;. \end{array}$$ (9)

Bacteria and phage co-exist if F<1 so that $b\bar{\eta }<1-\frac{\kappa (b(1-\alpha )-1)}{f(b-1)}$. This is an implicit expression because $\bar{\eta }$ itself depends on the distribution of bacteria with different spacers. The coexistence solution can be computed analytically

$$\begin{array}{cc} & gv=bfF\;,\\ & \frac{n_{i}}{n_{0}}=\alpha_{i}\;\frac{bfF}{\kappa -fF(1-\eta_{i}b)}\;,\\ & \frac{\sum_{i=1}^{N}n_{i}}{n_{0}}=\frac{b(1-\alpha )-1}{1-b\bar{\eta }}\;. \end{array}$$ (10)

We see that the spacer distribution depends on the acquisition and failure probabilities (*α*_*i*_ and *η*_*i*_). As discussed in the single spacer case, the third equation gives a way to measure the average failure probability ($\bar{\eta }$) of spacers by turning off the acquisition machinery after a diverse population of spacers is acquired [4, 28]. (This remains true even if the spacer also affects the growth rate—see S1 File). Given knowledge of the spacer failure probabilities (*η*_*i*_) from single spacer experiments, we can also obtain the acquisition probabilities (*α*_*i*_) by measuring the ratio of spacer enhanced to wild type bacteria (*n*_*i*_/*n*_0_) and using the second equation in (Eq 10).

**Fig 4:**
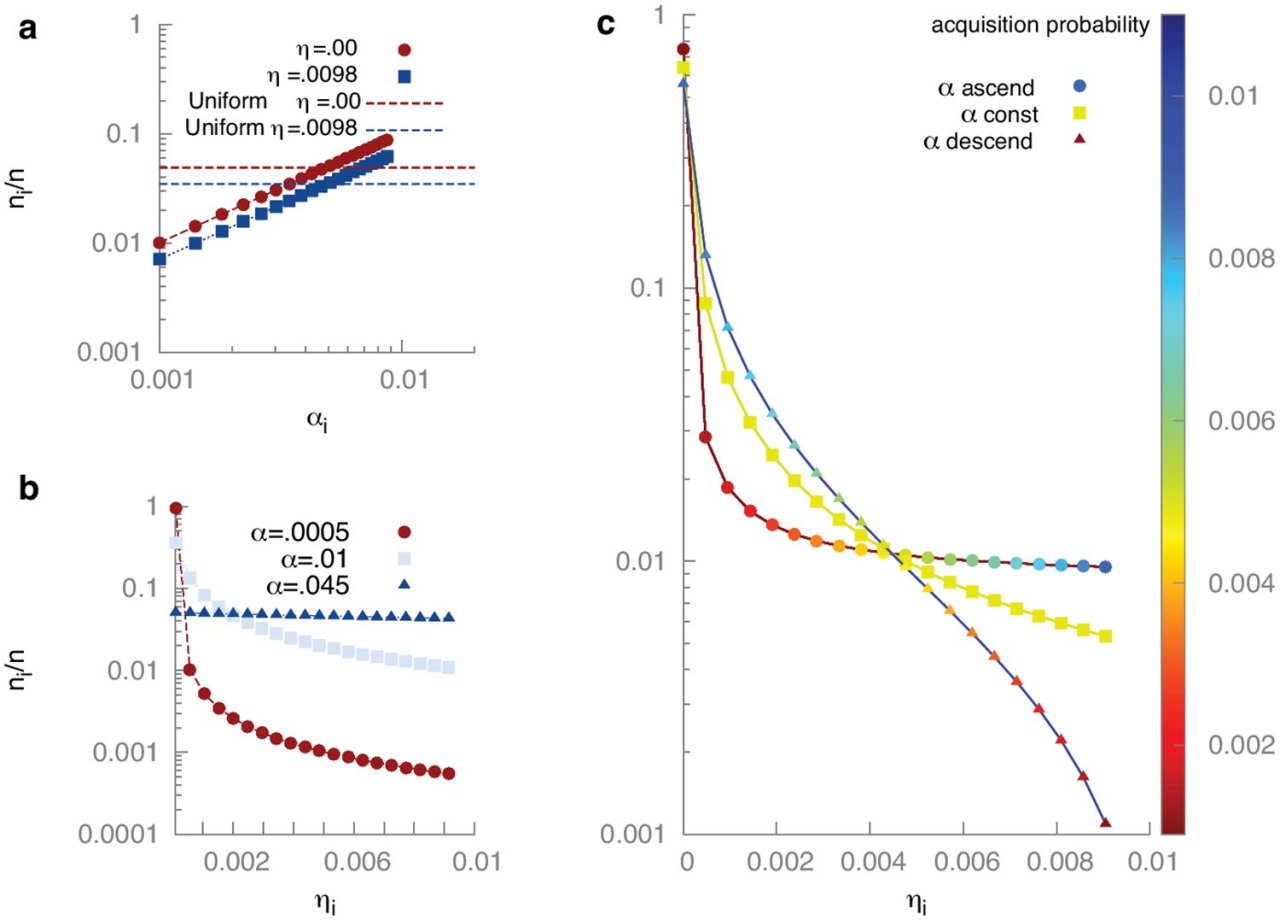
The distribution of bacteria with 20 spacer types. In these simulations, 100 phage are released upon lysis (burst size *b* = 100) and the carrying capacity for bacteria is *K* = 10^5^. All rates are measured in units of the bacterial growth rate *f*: the lysis rate is *μ*/*f* = 1, the phage adsorption rate is *g*/*f* = 10^−4^, the spacer loss rate is *κ*/*f* = 10^−2^. (Panel **a**) Distribution of spacers as a function of acquisition probability *α*_*i*_ given a constant failure probability *η*_*i*_ = *η*. (Eq 10) shows that the abundance depends linearly on the acquisition probability: *n*_*i*_/*n* ∝ *α*_*i*_/*α*. Horizontal lines give the reference population fraction of all spacers if they all have the same acquisition probability with the indicated failure probability *η*. (Panel **b**) Distribution of bacteria with different spacers as a function of failure probability *η*_*i*_ given a constant acquisition probability *α*_*i*_ = *α*/20. For small *α*, the distribution is highly peaked around the best spacer while for large *α* it becomes more uniform. (Panel **c**) The distribution of spacers when both the acquisition probability *α*_*i*_ and the failure probability *η*_*i*_ vary. The three curves have the same overall acquisition rate *α* = ∑_*i*_ *α*_*i*_ = .0972. The color of the dots indicates the acquisition probability and the *x*-axis indicates the failure probability of each spacer. When the acquisition probability is constant (green curve i.e. *α*_*i*_ = *α*/20) the population fraction of a spacer is determined by its failure probability. If the acquisition probability is anti-correlated with the failure probability (blue curve), effective spacers are also more likely to be acquired and this skews the distribution of spacers even further. If the acquisition probability is positively correlated with the failure probability (red curve), more effective spacers are less likely to be acquired. Despite this we see that the most effective spacer still dominates in the population.

The second equation in (Eq 10) also allows us to make qualitative predictions about mechanisms affecting the steady state spacer distribution. First, the steady state abundance of each spacer type is proportional to its probability of acquisition (*α*_*i*_). This implies that, if all else is kept fixed, a large difference in abundance can only come from a large difference in acquisition probability (see Fig 4a).

In contrast, the dependence on the failure probability (*η*_*i*_) appears in the denominator, so that large variations in abundance can follow from even modest differences in effectiveness (Fig 4b). When spacers differ in both acquisition and failure probability, the shape of the distribution is controlled mostly by the differences in effectiveness, with acquisition probability playing a secondary role (Fig 4c). This suggests that the distribution of spacers observed in experiments, with a few spacer types being much more abundant than the others [12], is likely indicative of differences in the effectiveness of these spacers, rather than in their ease of acquisition. The distribution of spacers as a function of ease of acquisition and effectiveness is shown for a larger number of spacers in S1 File (Fig D in S1 File), with the same qualitative findings.

Our model also predicts that the overall acquisition probability (*α*) is important for controlling the shape of the spacer distribution. Large acquisition probabilities tend to flatten the distribution, leading to highly diverse bacterial populations, while smaller acquisition probabilities allow the most effective spacers to take over (Fig 4b). This raises the possibility that the overall spacer acquisition probability of bacteria could be under evolutionary selection pressure as a means of trading off the benefits conferred by diversity in dealing with an open environment against the benefits of specificity in combatting immediate threats. This idea could be tested in directed evolution experiments where bacteria are grown in artificial environments with less or more variability in the phage population.

## Discussion

The CRISPR mechanism in bacteria is an exciting emerging arena for the study of the dynamics of adaptive immunity. Recent theoretical work has explored the co-evolution of bacteria and phage [18, 29, 30]. For example, Levin et al. [18] modeled several iterations of an evolutionary arms race in which bacteria become immune to phage by acquiring spacers, and the viral population escapes by mutation. Han et al. [29] studied coevolution in a population dynamics model in which there are several viral strains, each presenting a single protospacer modeled by a short bit string. Childs et al. [30] also used a population dynamics model to study the long-term co-evolution of bacteria and phage. In their model, bacteria can have multiple spacers and viruses can have multiple protospacers, and undergo mutations.

Our goal has been to model the effect of different properties of the spacers, such as their ease of acquisition and effectiveness, on their abundance in a setting where there is only enough time to acquire a single spacer. The reason for the latter restriction is that it leads to a more easily interpretable experimental setting. Our goal is not to study long-term bacteria-virus co-evolution, but rather to build a model of the early dynamics of CRISPR immunity that will allow experimentalists to extract key dynamical parameters from their data. An advantage of our model is that it allows study of regimes with a large number of spacer types. We aimed for a model with the minimal interactions that could explain existing observations, such as an over-abundance of a small number of spacers compared to the rest and the coexistence of phage and bacteria [12, 18, 20]. We are specifically interested in the possibility that encounters with a single phage could lead to the acquisition of diverse spacers [19], a phenomenon that could not be explained by the model of Han et al. [29]. Likewise, Levin et al. [18] did not explicitly model the spacer types and hence could not address their diversity. Furthermore, their model captured coexistence by postulating an as-yet-undetected lysis product from wild type bacteria that harms spacer enhanced ones. By contrast, we showed above that coexistence, in absence of any other mechanisms of immunity, can be obtained simply by including spacer loss, which has been experimentally observed [22, 27, 31].

Coexistence was also addressed by Haerter el al. [32] and Iranzo et al. [24]. Haerter et al. exploit spatial heterogeneity, while our model shows that coexistence can also occur in well-mixed populations. Coexistence in [24] occurs due to innate immunity for wild type bacteria. In the latter model, the CRISPR mechanism is taken to incur a cost to the bacteria, and thus loss of the CRISPR locus can occur as a consequence of competition between CRISPR and other forms of immunity, but is not an essential ingredient for coexistence. Their study also focused on longer timescales compared to our work. Childs et al. [30] discuss the possibility of coexistence, but only in the context of homogeneous bacterial populations, that are either all immune or all wild type. We show that coexistence of both immune and wild type bacteria with phage is possible given a nonzero rate of spacer loss. Finally, Weinberger et al. [33] used a population genetic model in which the sizes of the bacterial and phage populations are fixed, thus precluding study of the conditions required for coexistence. The model also did not consider potential differences in the efficacy of spacers.

Coexistence can also be obtained by placing the bacteria and phage in a chemostat or subjecting them to serial dilutions [16]. While in some ways this may be a better approximation for natural environments, in this work we focus on experimental conditions in which the interaction takes place in a closed environment. We predict that when dilution is negligible, spacer loss is necessary for the existence of a phase where wild-type bacteria, spacer-enhanced bacteria, and phage co-exist. When there is dilution, coexistence can occur without spacer loss [16], but we show in S1 File that this requires a difference in the growth rates of wild-type and spacer-enhanced bacteria. This difference is known to be small in general [12, 22], and hence the dilution mechanism for coexistence will lead to small viral populations at steady state which will be at risk of extinction due to stochastic variation. By contrast, coexistence through spacer loss can support robust steady state viral populations.

We have also addressed factors that influence the spacer distribution across the bacterial population. This issue was also studied in He et al. [34] and Han et al. [29], but they focused on the way in which diversity depends on position within the CRISPR locus as opposed to the properties of spacers that influence their relative abundance. Childs et al. [19, 30] were also interested in spacer diversity, but assumed that all spacers have similar acquisition probabilities and effectiveness, while we have sought precisely to understand how differences in these parameters affect diversity.

Our model makes several predictions that can be subjected to experimental test. First, spacer loss [22, 27, 31] is a very simple mechanism that allows for coexistence of bacteria and phage. In particular, spacer loss allows coexistence even in the absence of dilution, and permits robust steady state viral populations even if the growth rates of wild-type and spacer-enhanced bacteria are similar. Direct measurements of the rates of spacer loss may be possible, and would furnish an immediate test of our model. Alternatively, our model provides a framework for an indirect measurement of the spacer loss rate. Specifically, this rate is proportional to the viral population and the fraction of unused capacity at steady state. When the probability of spacer loss is small, our formalism predicts a correspondingly small average viral population.

Of course, the population in any given experiment experiences fluctuations which could lead to extinction. An interesting avenue for future work is to include such stochasticity, which would then predict the typical time-scale for viral extinction corresponding to a given probability of spacer loss. This time-scale can be compared with experimental observations [35]. A stochastic model of this dynamics was used by Iranzo et al. [24], but did not consider differences in spacer effectiveness. In order to check whether the result from a stochastic scenario would be different from what we found, we checked the stability of the deterministic solution with respect to initial conditions. The system is able to equilibrate in a reasonable time-scale suggesting that the deterministic solution is stable. This is an indication of robustness against stochastic fluctuations.

The effectiveness parameters in our model could be extracted in experiments where bacteria are engineered to have specific spacers [36] and acquisition is disabled [4, 28]. In principle the acquisition parameters could be measured by freezing bacterial populations soon after an infection, although initial conditions would require careful control. Once these parameters are measured, they can be plugged back into the full set of equations to make predictions for the CRISPR dynamics even in the case when acquisition is enabled. A comparison between the measured and predicted dynamics in the presence of CRISPR acquisition would constitute a test of our model. Alternatively, our model could be fit to measured dynamics to extract the parameters and then tested by comparing with the steady state.

When multiple protospacers are available, we showed that the acquisition probability linearly affects the steady state spacer distribution, while the proportion of more effective spacers is magnified by the dynamics. Thus, a highly peaked spacer distribution as seen in some studies [12] is more likely to occur because of differences in effectiveness if protospacers are acquired with roughly equal probabilities. In fact, it does seem that some genomic sequences are acquired more frequently than others [6, 13]. While the mechanism for this enhancement has not been fully clarified, one possibility is that the more commonly acquired sequences are simply those that are less prone to mutation in the viral genome. This could be tested by sequencing the virus together with the CRISPR-cassettes in a co-evolving population of bacteria and phage. This mechanism for enhancing acquisition probability of some spacers is readily incorporated in our model.

Various extensions of our model are possible. For example, in describing longer timescale experiments we can include the fact that CRISPR cassettes can contain many spacers [34]. Furthermore, we could include the possibility of “priming” where the presence of some spacers increases the probability of acquiring others [6]. Such an effect would introduce correlations between different spacer populations *n*_*i*_ and *n*_*j*_ that can be tested experimentally.

Our model showed that high acquisition probabilities will lead to greater diversity in the spacer distribution, while strong selection will tend to homogenize the population of spacers in favor of the most effective one for the current threat. This suggests that bacteria should adapt the overall spacer acquisition probability to the amount of viral diversity in their environment, perhaps by transcriptional regulation of the *cas* genes. Given an appropriate fitness function and viral landscape our modeling framework could be used to predict the optimal acquisition probability.

## Supporting information

S1 file Supporting information. (PDF)
