# Supplementary material for: Dynamics of adaptive immunity against phage in bacterial populations
Source: PLoS Comput Biol. 2017 Apr 17;13(4):e1005486. doi: 10.1371/journal.pcbi.1005486 (PMC5411097; doi:10.1371/journal.pcbi.1005486)
Supplement: S1 file — (PDF) [file pcbi.1005486.s001.pdf]

Supplementary Information

Dynamics of adaptive immunity against phage in bacterial populations

Contents

1 Materials and Methods **1**

2 Detailed dynamics and steady state with one type of spacer **1**

2.1 Transient behavior 1

2.2 Steady state solutions and stability analysis 2

3 Extensions of the model **6**

3.1 Viral decay 7

3.2 The dilution regime 8

4 Detailed steady state solution for multiple spacers **10**

1 Materials and Methods

We numerically integrated our population dynamics equations using custom code written in C. The order of magnitude of the burst size b, carrying capacity K and growth rate f were taken from Childs et al. (2012), the spacer loss rate κ was estimated from Jiang et al. (2013), and the spacer failure probability η and acquisition probability α were variables that we scanned over. We took the death rate μ of infected bacteria to be comparable to the growth rate f. To estimate the order of magnitude of the phage adsorption rate g we used a simple argument based on diffusion. Because of the large difference in size, we approximate phage particles with points and focus on a single bacterial cell, modeled as a perfectly absorbing sphere of radius R. Fick's second law, $\dot{c} = D\Delta c$, can be used to calculate the concentration profile of phage around the bacteria at stationarity, leading to $c(r) = c_p\big(1 - \frac{R}{r}\big)$, where c_p is the concentration of phage far away from bacteria. Fick's first law then gives us the flux at the sphere, whose integral gives the rate at which phage are absorbed, $4\pi D c_p R$, where D is the diffusion coefficient. Using Einstein's relation $D = \frac{k_B T}{6\pi\eta r_v}$ to estimate the diffusion coefficient (with η the dynamic viscosity of the medium and r_v the size of the virus), we get that an estimate for g is

$$g = \frac{2}{3V}\frac{k_b T}{\eta}\frac{R}{r_v}\,, \tag{1}$$

where V is the experimental volume, which only appears here because we defined g in terms of particle numbers instead of concentrations. Using $V \sim 1\,\mu\text{L}$, $\eta = 8\times10^{-4}\,\text{Pa}\cdot\text{s}$ (for water at 30°), $T = 300\,\text{K}$, $R \sim 1\,\mu\text{m}$, $r_v \sim 0.1\,\mu\text{m}$, we get $g \sim 10^{-4}\,\text{hour}^{-1}$. This is very close to experimental values observed in in vitro experiments of bacteria and phage Weld et al. (2004).

2 Detailed dynamics and steady state with one type of spacer

2.1 Transient behavior

In addition to the numerical studies presented in the main text, some aspects of the dynamics of our model can be described analytically. This allows us to get further insights into general features of the solutions and

how these features depend on the parameters.

Let us analyze the initial trend in the bacterial population in the single protospacer model (eq. **[1]** in the main text):

$$\begin{aligned}
\dot{n}_0 &= f_0 \left(1 - \frac{n}{K}\right) n_0 + \kappa n_1 - g v n_0 \,, \\
\dot{n}_1 &= f_1 \left(1 - \frac{n}{K}\right) n_1 - \kappa n_1 - \eta g v n_1 + \alpha \mu I_0 \,, \\
\dot{I}_0 &= g v n_0 - \mu I_0 \,, \\
\dot{I}_1 &= \eta g v n_1 - \mu I_1 \,, \\
\dot{v} &= b\,(1-\alpha)\,\mu I_0 + b \mu I_1 - g v (n_0 + n_1) \,.
\end{aligned} \tag{2}$$

At the time of inoculation, the population is entirely wild type, so $n_1(0) = 0$, and there are no infected bacteria, $I_0(0) = 0$, $I_1(0) = 0$. The total bacterial population is $n(0) \equiv n_0(0) < K$. The dynamics of $n(t)$ are governed by eq. **[2]** from the main text,

$$\dot{n} = f_0 (n_0 + r n_1) \left(1 - \frac{n}{K}\right) - \mu (1-\alpha) I_0 - \mu I_1 \,, \tag{3}$$

which implies that in this case $\dot{n}(0) > 0$, *i.e.,* the bacteria always start off with growth. This is a result of the latency between viral infection and death. However, we expect this growth to be short lived: after a time of order μ^{-1}, the infected cells should start dying and the bacterial population should go down. It turns out that growth can sometimes last much longer than that, as shown below.

Suppose we have $t \ll 1/\mu$ and, for simplicity, assume $n_0(0) \ll K$; from the system of equations (2) we get

$$\begin{aligned}
n_0 &= n_0(0) + \big[f_0 - g v(0)\big] n_0(0)\, t + \mathcal{O}(t^2) \,, \\
I_0 &= g v(0) n_0(0)\, t + \mathcal{O}(t^2) \,.
\end{aligned} \tag{4}$$

Similarly $n_1 = \mathcal{O}(t^2)$ and $I_1 = \mathcal{O}(t^2)$, implying that

$$\dot{n} = f_0 n_0(0) \left\{ 1 - t \left[g v(0) \left(1 + \frac{\mu(1-\alpha)}{f_0} \right) - f_0 \right] \right\} + \mathcal{O}(t^2) \,. \tag{5}$$

If $gv(0) > f_0$ then growth ends in a time

$$\begin{aligned}
t_p &= \frac{1}{g v(0) \left(1 + \frac{\mu(1-\alpha)}{f_0}\right) - f_0} < \frac{f_0}{\mu(1-\alpha) g v(0)} \\
&< \frac{1}{\mu(1-\alpha)} \approx \frac{1}{\mu} \,,
\end{aligned} \tag{6}$$

assuming that the acquisition probability α is small. Conversely, if

$$g v(0) < \frac{f_0}{1 + \mu(1-\alpha)/f_0} \,, \tag{7}$$

the initial growth of the wild type bacteria continues past the initial latency time $t = 1/\mu$. This prediction of the model can be tested via optical density measurements. Of course, past this time, the approximations we made above no longer hold; eventually the wild type population will decline, being overtaken by the virus, and the bacteria will go through a bottleneck; see Fig. 3 of the main text. Recovery from this bottleneck is due to CRISPR spacer acquisition.

2.2 Steady state solutions and stability analysis

Coexistence solution: $v \neq 0$ and $n \neq 0$. The steady state solutions for the system in eq. (2) are obtained by setting all the derivatives to zero. Solving the equations for the number of infected bacteria and plugging

these into the equation for the viral dynamics, we get

$$
\begin{aligned}
I_0 &= \frac{g}{\mu} v n_0 \,, \\
I_1 &= \frac{g\eta}{\mu} v n_1 \,, \\
n_1 &= \frac{b(1-\alpha)-1}{1-b\eta} n_0 \,,
\end{aligned}
\tag{8}
$$

where we assumed $v \neq 0$.

Since I_i, n_i, and v are population numbers, they must be non-negative. From eq. (8) above, this requires that $b\eta < 1$ and $b\alpha < b - 1$[1]. If these conditions are not met, the co-existence solution is not feasible, and we get either $n_i = I_i = 0$ or $v = 0$.

The remaining steady state values can be obtained after some tedious but straightforward algebra:

$$
\begin{aligned}
n &= K(1-\mathcal{F}_r) \,, \\
\mathcal{F}_r &= \frac{\kappa}{f_0} \frac{b(1-\alpha)-1}{(1-b\eta)\left(b-1+(r-1)\dfrac{b(1-\alpha)-1}{1-\alpha-\eta}\right)} \,, \\
gv &= f_0 \mathcal{F}_r + \kappa \, \frac{b(1-\alpha)-1}{1-b\eta} \,, \\
\frac{n_0}{n} &= \frac{\mu(1-b\eta)}{b\mu(1-\alpha-\eta) + \left(f_0 \mathcal{F}_r + \kappa \, \dfrac{b(1-\alpha)-1}{1-b\eta}\right)(1-\eta-\eta b\alpha)} \,, \\
\frac{n_1}{n} &= \frac{\mu(b(1-\alpha)-1)}{b\mu(1-\alpha-\eta) + \left(f_0 \mathcal{F}_r + \kappa \, \dfrac{b(1-\alpha)-1}{1-b\eta}\right)(1-\eta-\eta b\alpha)} \,,
\end{aligned}
\tag{9}
$$

where we see that the virus concentration at steady state is proportional to the rate of spacer loss (κ). Here the notation $\mathcal{F}_r$ is used to emphasize the fact that we allow for different growth rates for wild type and spacer enhanced bacteria.

Compared to the expression from eq. **[3]** in the main text, the fraction of unused capacity ($\mathcal{F}$) changes when the wild type and spacer enhanced growth rates are unequal. The magnitude of the change obeys

$$
\frac{\mathcal{F}_r}{\mathcal{F}} = \frac{1}{1 + \gamma \, \dfrac{b(1-\alpha)-1}{(b-1)(1-\alpha-\eta)}} = 1 - \gamma \, \frac{b(1-\alpha)-1}{(b-1)(1-\alpha-\eta)} + \mathcal{O}(\gamma^2) \,,
\tag{10}
$$

where $\gamma = r - 1$.

The positivity of n_0 and n_1 also implies that $\mathcal{F}_r < 1$. This translates into a more stringent condition on the failure probability of the spacer (η). For bacteria to be able to resist infection and for the co-existence solution to be feasible, we need $\eta < \eta_c$, where the critical failure probability is

$$
\eta_c = \frac{1}{b}\left(1 - \frac{\kappa}{f_0}\frac{b(1-\alpha)-1}{b-1}\right) + \gamma \, \frac{\kappa}{f_0} \frac{b(1-\alpha)-1}{(b-1)(b-1+\kappa/f_0)} - \gamma^2 \, \frac{\kappa}{f_0} \frac{b\big(b(1-\alpha)-1\big)}{(b-1+\kappa/f_0)^3} + \mathcal{O}(\gamma^3) \,.
\tag{11}
$$

This gives the next couple of terms in the expansion in eq. **[4]** in the main text. We write this as an expansion in $\gamma = r - 1$ because experiments suggest that differences between the growth rates of wild type and spacer enhanced bacteria are small.

[1] In principle the opposite conditions could also hold, $b\eta > 1$ and $b\alpha > b - 1$, implying $\alpha > (b-1)/b$. However, in realistic conditions, b is large (of order 100 or 1000), and so $(b-1)/b \approx 1$, while α is typically much smaller than 1.

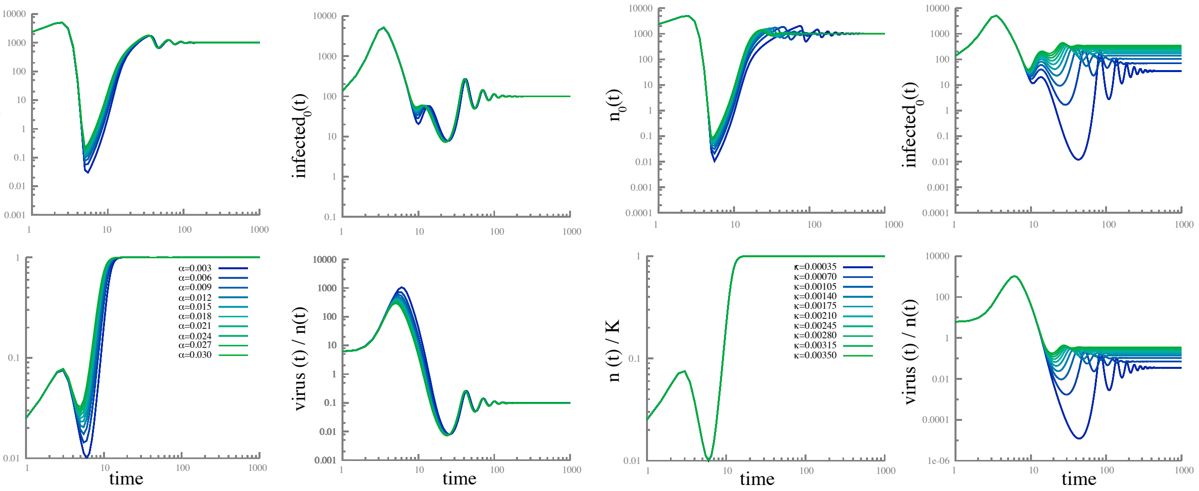

Fig A: Time-dependence of the population of wild type bacteria, both growing (n_0) and infected (I_0), the fraction of carrying capacity occupied by bacteria (n/K), and the viral population (v) for various choices of acquisition probability (α; left panel) and rate of spacer loss (κ; right panel). Both axes are on a log-scale because the dynamic range varies significantly between the early and late periods. Other parameters are chosen similar to the main text: the growth rates of spacer enhanced and wild type bacteria are equal ($r = 1$), the phage adsorption rate is given by $g/f_0 = 10^{-5}$, the death rate of infected bacteria is given by $\mu/f_0 = 1$, the burst factor is $b = 100$, and the spacer failure probability $\eta = 0$. The initial population is all wild type $n = n_0 = 1000$ and maximum capacity $K = 10^5$, and the initial multiplicity of infection (MOI; v/n) is 10 such that $v(0) = 10^4$. In the left panel, the rate of spacer loss is kept fixed at $\kappa/f_0 = 0.001$. In the right panel, the acquisition probability is kept fixed at $\alpha = 0.003$. We see that the oscillations in the wild type bacteria and viral populations are more strongly damped for large spacer loss rates.

Another interesting limit to consider is when the burst size b is very large, which is the case for typical viruses. When $b \to \infty$, the product between the burst size and the critical failure probability simplifies to

$$b\eta_c = 1 - \frac{\kappa(1-\alpha)}{f_1} + \mathcal{O}(b^{-1})\,. \tag{12}$$

This means that, apart from being inversely proportional to the burst factor, the critical failure rate at large b only depends on the rate of spacer loss (κ), the acquisition probability (α), and the growth rate of the spacer enhanced bacteria (f_1). In particular, it does not depend on the wild type growth rate.

A numerical study shows that when the coexistence solution exists ($\eta < \eta_c$), it is also stable for a wide range of parameters. Altering the spacer acquisition probability (α), the rate of spacer loss (κ), or the failure probability (η) in a wide range does not preclude the coexistence state, although it can lead to significant changes in the population dynamics (see Fig. A and Fig. B). Interestingly, the dynamics of the total number of bacteria (n) is almost insensitive to the rate of spacer loss (κ). The viral population, and therefore also the population of infected bacteria, are much more strongly affected by changes in the rate of spacer loss. Frequent loss of spacers leads to a stronger damping and shorter period for the oscillations in the viral population, while less frequent loss greatly enhances the amplitude of these oscillations.

The ability to acquire spacers has a large effect on the early dynamics of the bacterial population, while not greatly affecting the viral dynamics. Lower acquisition probabilities lead to a tighter bottleneck for the bacteria, as they require a longer time to gain the CRISPR immunity that they need to fight viral infection.

The spacer failure probability (η) does not affect the total bacterial population too much, but has a large effect on the steady state population of phage and wild type bacteria, as well as on the transient dynamics leading to the steady state. More effective spacers lead to larger numbers of wild type bacteria and fewer viruses, shorter transients, and more oscillatory dynamics. In contrast, bacteria with less effective spacers (larger η) can take a long time to reach steady state, don't seem to exhibit oscillations, and lead to fewer wild type bacteria and more viruses.

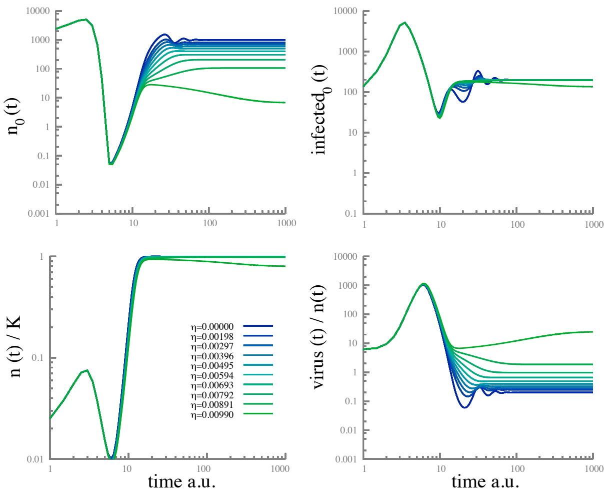

Fig B: Time-dependence of the population of wild type bacteria, both growing (n_0) and infected (I_0), the fraction of carrying capacity occupied by bacteria (n/K), and the viral population (v) for various choices of the spacer failure probability (η). Both axes have a log scale to better reveal the dynamical range. Other parameters are chosen similar to the main text: the growth rates of spacer enhanced and wild type bacteria are equal ($r = 1$), the phage adsorption rate is given by $g/f_0 = 10^{-5}$, the death rate of infected bacteria is given by $\mu/f_0 = 1$, the burst factor $b = 100$, the rate of spacer loss is $\kappa/f_0 = 0.002$ and acquisition probability $\alpha = 0.003$. The initial population is all wild type, $n(0) = n_0(0) = 10^3$ and the initial multiplicity of infection (MOI; v/n) is 10 that means $v(0) = 10^4$.

When stochastic effects are taken into consideration, random fluctuations could lead to extinction of either bacteria or phage when these go through bottlenecks. Thus, smaller acquisition probabilities lead to higher chances of bacterial extinction, while smaller rates of spacer loss would be dangerous for the phage's survival. Very effective spacers also increase the oscillations in the viral dynamics, while also lowering the steady state viral population, so they too can contribute to viral extinction.

Virus extinction $v = 0$. When the virus goes extinct ($v = 0$), the steady state populations of wild type and spacer enhanced bacteria can depend on the initial conditions. We can gain some intuition into this case by considering a model in which the virus has already gone extinct; thus, $v = 0$ and $I_0 = I_1 = 0$. In this case, the system of equations simplifies to

$$\begin{aligned} \dot{n}_0 &= f_0 \left(1 - \frac{n}{K}\right) n_0 + \kappa n_1 \,, \\ \dot{n}_1 &= f_1 \left(1 - \frac{n}{K}\right) n_1 - \kappa n_1 \,, \end{aligned} \tag{13}$$

If we assume that the wild type and spacer enhanced growth rates are equal ($f_1 = f_0$), the solution can be found analytically:

$$\begin{aligned} n(t) &= \frac{K n(t_0)\, e^{f_0 (t-t_0)}}{K - n(t_0)\big(1 - e^{f_0(t-t_0)}\big)} \,, \\ \frac{n_1(t)}{n(t)} &= \frac{n_1(t_0)}{n(t_0)}\, e^{-\kappa (t-t_0)} \,, \\ \frac{n_0(t)}{n(t)} &= 1 - \frac{n_1(t)}{n(t)} \,, \end{aligned} \tag{14}$$

where t_0 is the initial time and $n(t_0)$ and $n_1(t_0)$ are the initial total population of bacteria and the initial population of spacer enhanced bacteria, respectively. In the long term limit the bacterial population reaches

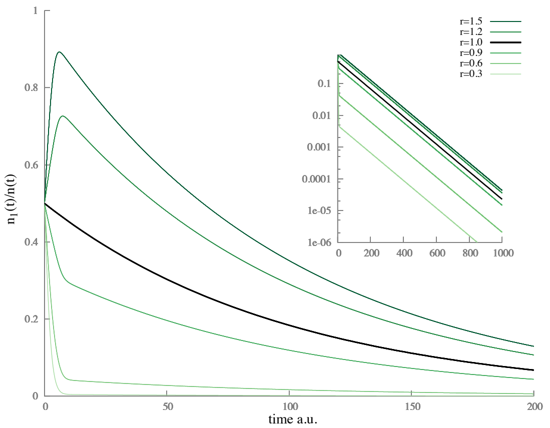

Fig C: Dynamics of the fraction of spacer enhanced bacteria in absence of virus. The plots show the numerical solution of the system of equations given in (13) when $K = 10^5$ and spacer enhanced and wild type growth rates are different ($r = f_1/f_0 \neq 1$). The initial values $n_1(0) = n_0(0) = 50, n(0) = 100$ and $\kappa = .01$. For comparison, we show the analytical solution reported in eq. (14) for the special case $r = 1$ (black line). The long-term decay of the spacer-enhanced population relative to wild type is simply due to spacer loss in the absence of virus. In the inset we emphasize the exponential decays for longer time scales by using a log scale.

the carrying capacity, $n(t) \to K$. If there is spacer loss ($\kappa \neq 0$), the spacer enhanced bacteria eventually disappear, so the steady state in this case is $n_0 = n = K$, $n_1 = 0$, independently of initial conditions. If there is no spacer loss ($\kappa = 0$), the fraction of bacteria that are spacer enhanced stays constant as the bacteria grow to capacity.

We can use this result to understand what happens in the more general case when the viral population starts off non-zero but eventually dies out ($v \to 0$). In this case we need to compute the viral extinction time $t_0 = t_{\text{eff}}$ such that $v(t_{\text{eff}}) \sim 0$. The arguments above suggest that viral extinction can only happen if there is no spacer loss – this is because even an exponentially small number of viruses will be able to multiply if presented with wild-type bacteria. Assuming the spacer loss rate is zero, the fraction of the total bacterial population that contains spacers will stay approximately constant from the time of viral extinction.

We can also investigate what happens when the spacer enhanced growth rate differs from that of the wild type, but this requires numerical simulations (Fig. C). Just like in the case when the growth rates are equal, the population of spacer-enhanced bacteria $n_1(t)$ decays exponentially to zero at large times.

In summary, when bacteria can lose spacers and the failure probability is lower than the critical value from eq. (11), virus and bacteria co-exist in a steady state after the initial transient dynamics.

3 Extensions of the model

In realistic situations, bacteria and viruses undergo decay even in the absence of any external threats. We can incorporate this into our model by adding decay terms into the system of equations (2):

$$
\begin{aligned}
\dot{n}_0 &= f_0 \left(1 - \frac{n}{K}\right) n_0 + \kappa n_1 - g v n_0 - s n_0 \,, \\
\dot{n}_1 &= f_1 \left(1 - \frac{n}{K}\right) n_1 - \kappa n_1 - \eta g v n_1 + \alpha \mu I_0 - s n_1 \,, \\
\dot{I}_0 &= g v n_0 - (\mu + s) I_0 \,, \\
\dot{I}_1 &= \eta g v n_1 - (\mu + s) I_1 \,, \\
\dot{v} &= b\,(1 - \alpha)\, \mu I_0 + b \mu I_1 - g v (n_0 + n_1) - s' v \,.
\end{aligned}
\tag{15}
$$

Here s is the decay rate for bacteria, assumed to be the same regardless whether they are infected or not and whether they are spacer enhanced or wildtype, while s' is the decay rate for phage.

The formalism above can also be used to model a rather different phenomenon: the dynamics of a mixture of bacteria and phage in the case where the experimental preparation includes serial dilutions or chemostats. In these cases, the bacterial culture is either periodically or continuously removed and added to fresh sterile medium. This leads to a continual renewal of nutrients that allows the bacterial population to keep growing. If natural decay is negligible compared to dilution, we can set $s' = s$ in eqs. (15).

To simplify the analysis, we look at two cases: viral decay without bacterial decay, and dilution. The general case can be treated similarly.

3.1 Viral decay

We start with the case in which viruses decay at a rate s', but there is no bacterial decay ($s = 0$). We look for steady state solutions for eqs. (15), with the added simplification of ignoring spacer loss, $\kappa = 0$. We will generally assume that the growth rates of spacer enhanced and wild type bacteria are similar, in accordance with experiments. We will thus write $f_1 = f_0(1 + \gamma)$ and perform an expansion in γ. These approximations greatly simplify the analytical manipulations without changing the qualitative picture for moderate variations in parameters, as we checked numerically.

There are three types of steady state solutions: 1) a coexistence scenario where spacer enhanced bacteria, wild type bacteria, and virus coexist; 2) a monoclonal-bacteria scenario where wild type bacteria go extinct leaving only spacer enhanced; and 3) an infection-free scenario where viruses go extinct.

1. The coexistence scenario.

   The steady state solutions expanded to first order in $\gamma = (f_1 - f_0)/f_0$ are

$$\begin{aligned} n_0 &= \frac{s'(1-\eta)}{g(b-1)(1-\alpha-\eta)} + \frac{s'\alpha(1-b\eta)}{g(b-1)^2(1-\alpha-\eta)^2}\,\gamma + O(\gamma^2)\,, \\ n_1 &= \frac{-s'\alpha}{g(b-1)(1-\alpha-\eta)} + \frac{s'\alpha\big(b(1-\alpha)-1\big)}{g(b-1)^2(1-\alpha-\eta)^2}\gamma + O(\gamma^2)\,, \\ v\ &= \frac{f_0\mu(1-\alpha-\eta)\big((b-1)gK - s'\big)}{g\big[(b-1)gK\mu(1-\alpha-\eta) + f_0 s'(1-\alpha\eta-\eta)\big]} + O(\gamma)\,. \end{aligned} \tag{16}$$

   Typically, acquisition and failure probabilities are not large, so that we can expect $\alpha + \eta < 1$. This implies that when the difference in growth rate, γ, is small, n_1 in the solution above is formally negative. Physically, this means that the coexistence scenario is not feasible in the biologically-plausible region of parameters.

2. The monoclonal-bacteria scenario.

   This scenario is characterized by the absence of wild type

$$n_0 = 0\,, \qquad n_1 = \frac{s'}{g(b\eta - 1)}\,, \qquad v = \frac{f_1\mu\big[gK(b\eta-1) - s'\big]}{g\eta\big[gK\mu(b\eta-1) + f_1 s'\big]}\,. \tag{17}$$

   This solution exists only when the failure rate is high $\eta > 1/b$, because n_1 must be positive. This regime is opposite to the one we considered in the main text, where we showed that the bacterial population can survive only when the failure probability η is small enough to compensate for the infection bursting factor, $\eta < 1/b$. The non-zero viral decay rate acts in favor of the bacteria, giving them a chance to survive even when the failure probability is high. If spacers can be lost (i.e. $\kappa \neq 0$), then some of the surviving spacer-enhanced bacteria will revert to wild type, thus maintaining a diverse population. This confirms from a different perspective that spacer loss plays a key role in establishing coexistence of the virus with both the spacer-enhanced and the wild-type bacteria.

3. The infection-free scenario.

   In this case, the viral infection is completely cleared, and we get

$$n_0 = K - n_1\,, \qquad n_1 = K\,\frac{b(1-\alpha)-1}{b(1-\alpha-\eta)} - \frac{s'}{bg(1-\alpha-\eta)}\,, \qquad v \to 0\,. \tag{18}$$

This is the case where the bacterial population is able to cope with the infection and grows up to maximum capacity. The main difference with respect to the absence of viral decay rate, $s' = 0$, is that wild type reaches a higher value proportional to the rate s'.

3.2 The dilution regime

In the case of either serial dilutions or chemostat conditions, if we ignore natural decay for bacteria and phage, the system dilutes both populations at the same rate, $s' = s$. As above, we start by neglecting spacer loss, $\kappa = 0$, and consider conditions in which spacer enhanced and wild type bacteria have almost equal growth rates, so that we can expand in $\gamma = (f_1 - f_0)/f_0$.

We first consider the dynamics in the limit in which the infected bacteria are killed instantaneously, $\mu \to \infty$. In this case, the system of equations simplifies to

$$\begin{aligned} \dot{n}_0 &= f_0 \left(1 - \frac{n}{K}\right) n_0 - gvn_0 - sn_0 \,, \\ \dot{n}_1 &= f_1 \left(1 - \frac{n}{K}\right) n_1 - \eta gvn_1 + \alpha gvn_0 - sn_1 \,, \\ \dot{v} \ &= b\,(1-\alpha)\, gvn_0 + bgv\eta n_1 - gv(n_0 + n_1) - sv \,. \end{aligned} \tag{19}$$

If we consider the case in which infected bacteria do not instantaneously die, the steady state solutions change quantitatively, but not qualitatively.[2]

As before, we can identify three classes of steady state solutions.

1. The coexistence scenario.

   Here we get that

$$\frac{n_1}{n} = -\frac{\alpha}{1-\alpha-\eta} + \frac{\alpha f_0((b-1)gK - s)}{\big[f_0\big((b-1)gK - s\big) - (b-1)gKs\big](1-\alpha-\eta)^2}\,\gamma + O(\gamma^2)\,. \tag{20}$$

   As above, we expect that $\gamma \ll 1$ and that $\alpha + \eta < 1$ – under these conditions the positivity of n_1 implies that the coexistence solution is not feasible.

2. The monoclonal-bacteria scenario.

   Here wild type bacteria are absent:

$$\begin{aligned} n_0 &= 0\,, \\ n = n_1 &= \frac{s}{g(b\eta - 1)}\,, \\ gv\eta &= f_1 \left(1 - \frac{s}{gK(b\eta-1)}\right) - s\,. \end{aligned} \tag{21}$$

   This solution is feasible when $\eta > 1/b$, a regime where failure rate is high, opposite to the situation considered in the main text. The virus only survives because the spacer fails with a relatively high probability. However, if the dilution is large ($s > gK(b\eta - 1)$) there will be no viruses left at steady state because growth of virus in the bacteria is more than compensated for by loss due to dilution. Again in this regime adding spacer loss will keep wild type bacteria alive, allowing for coexistence of the two species of bacteria and the virus.

3. Low infection scenario.

   The only scenario in which virus and both types of bacteria co-exist without spacer loss is one where the spacer-enhanced and wild-type bacteria grow at different rates ($\gamma \neq 0$). The steady state solutions

[2] For example, consider the condition on the failure probably $b\eta = 1$ that defines the boundary of the region where steady state solutions exist. If the infected bacteria survive for a period of time before dying, it turns out that this condition will be replaced by $b\eta = w$ where $w = \frac{\mu+s}{\mu}$. The dimensionless parameter w effectively rescales the time of phage release from infected cells.

at leading order in γ are

$$\begin{aligned}
\frac{n_1}{n} &= x_1^{(0)} + \gamma x_1^{(1)} + O(\gamma^2)\,, \\
n_0 &= n - n_1\,, \\
n &= K\left(1 - \frac{s}{f_0}\right) + \gamma x_1^{(1)} \frac{bgK^2(1-\alpha-\eta)(f_0-s)^2}{f_0^2 s} + O(\gamma^2)\,, \\
v &= -\frac{s\big[gK(f_0-s)\big(b(1-\alpha)-1\big) - f_0 s\big]}{g(1-\alpha-\eta)\big[gK(b-1)(f_0-s) - f_0 s\big]}\gamma + O(\gamma^2)\,,
\end{aligned} \tag{22}$$

where the two expansion coefficients for the fraction of spacer enhanced bacteria are

$$\begin{aligned}
x_1^{(0)} &= \frac{b(1-\alpha)-1}{b(1-\alpha-\eta)} - \frac{f_0 s}{bgK(f_0-s)(1-\alpha-\eta)}\,, \\
x_1^{(1)} &= f_0 s^2 \frac{gK(f_0-s)\big(b(1-\alpha)-1\big) - f_0 s}{bgK\big[gK(b-1)(f_0-s) - f_0 s\big](1-\alpha-\eta)^2(f_0-s)^2}\,.
\end{aligned} \tag{23}$$

The number of viruses is proportional to the relative difference in growth rates, γ, so it is different from zero only when spacer enhanced and wild type bacteria grow at different rates.

In order to analyze the parameters where the solution is feasible, we further expand in the dilution parameter s, and get

$$x_1^{(0)} = \frac{b(1-\alpha)-1}{b(1-\alpha-\eta)} - \frac{s}{bgK(1-\alpha-\eta)} + O(s^2)\,, \qquad x_1^{(1)} = O(s^2)\,. \tag{24}$$

This means that for small dilution, the total bacterial population is not resource-limited, but there is a correction due to dilution. The coexistence between bacteria and virus is controlled by the relative difference in growth rate, γ, and the dilution, s:

$$\begin{aligned}
n &\approx K\left(1 - \frac{s}{f_0} + \gamma s\,\frac{b(1-\alpha)-1}{f_0(b-1)(1-\alpha-\eta)}\right)\,, \\
n_1 &\approx K\,\frac{b(1-\alpha)-1}{b(1-\alpha-\eta)} - s\,\frac{gK\big(b(1-\alpha)-1\big)+f_0}{f_0 bg(1-\alpha-\eta)} + \gamma s\,K\,\frac{\big(b(1-\alpha)-1\big)^2}{bf_0(b-1)(1-\alpha-\eta)^2}\,, \\
v &\approx -\gamma s\,\frac{b(1-\alpha)-1}{g(b-1)(1-\alpha-\eta)}\,.
\end{aligned} \tag{25}$$

For this solution to be feasible, we need $v > 0$. If the spacer enhanced bacteria have a higher growth rate than the wild type, $\gamma > 0$, this can only happen if $\alpha > 1 - 1/b$, implying an unrealistically high (close to unity) acquisition probability. The more realistic scenario occurs when the spacer enhanced bacteria grow slower than the wild type, $\gamma < 0$. In this case, coexistence between both bacterial species and phage can be observed, but the amount of phage is small, since it is proportional to the product of the relative difference in growth rates and the dilution rate, both of which are typically small.

Notice that the solutions we have found are not fundamentally new. Rather, they represent small corrections to the solutions we found without dilution. In the main text we found that coexistence of the phage with both bacterial species was enabled by spacer loss which then also implied that the bacterial population did not reach capacity. Here we see that dilution provides an alternative mechanism (other than spacer loss) for these effects, but only if spacer-enhanced and wild-type bacteria grow at different rates ($\gamma \neq 0$). Since this rate difference is measured to be small, and since dilution in typical experiments is an order of magnitude smaller than growth rate, we can conclude that the latter scenario for coexistence leads to small viral populations. Stochastic effects are then likely to lead to extinction of the virus. The spacer-loss mechanism discussed in the main text can lead to more robust viral populations at co-existence.

4 Detailed steady state solution for multiple spacers

In the main text, we showed the steady state values for the case of multiple spacers only when all the growth rates were the same. Here we generalize the dynamics from eq. [**6**] in the main text to the case where each spacer (i) has a different growth rate f_i,

$$\begin{aligned}
\dot{n}_0 &= f_0\left(1-\frac{n}{K}\right)n_0+\kappa\sum_{i=1}^{N}n_i-gvn_0\,,\\
\dot{n}_i &= f_i\left(1-\frac{n}{K}\right)n_i-\kappa n_i-\eta_i gvn_i+\alpha_i\mu I_0\,,\\
\dot{I}_0 &= gvn_0-\mu I_0\,,\\
\dot{I}_i &= \eta_i gvn_i-\mu I_i\,,\\
\dot{v}\ &= b\Big(1-\sum_{i=1}^{N}\alpha_i\Big)\mu I_0+b\mu\sum_{i=1}^{N}I_i-gv\Big(n_0+\sum_{i=1}^{N}n_i\Big)\,.
\end{aligned} \tag{26}$$

Setting the time derivatives to zero, we obtain

$$\begin{aligned}
I_0 &= \frac{g}{\mu}vn_0\,,\\
I_i &= \frac{g\eta_i}{\mu}vn_i\,,\\
\frac{\sum_i n_i}{n_0} &= \frac{b(1-\alpha)-1}{1-b\bar{\eta}}\,,
\end{aligned} \tag{27}$$

where the average failure probability ($\bar{\eta}$) is defined as in the main text, $\bar{\eta}=\sum\eta_i n_i/\sum n_i$ (eq. [**8**]). Similar to the case of a single spacer, we see that the ratio between the total number of spacer enhanced bacteria and the number of wild type bacteria is independent of the growth rates (f_i).

By introducing the growth rate ratio ($r_i=f_i/f_0$) and the average growth rate ratio

$$r=\frac{\sum_{i=1}^{N}r_in_i}{\sum_{i=1}^{N}n_i}\,. \tag{28}$$

we can now obtain the fraction of unused capacity ($\mathcal{F}_r=1-n/K$):

$$\mathcal{F}_r=\frac{\kappa}{f_0}\frac{b(1-\alpha)-1}{(1-b\bar{\eta})\left(b-1+(r-1)\dfrac{b(1-\alpha)-1}{1-\alpha-\bar{\eta}}\right)}\,, \tag{29}$$

which generalizes eq. [**8**] in the main text. The remaining steady state values are given by:

$$\begin{aligned}
n\ &= K(1-\mathcal{F}_r)\,,\\
gv &= f_0\mathcal{F}_r+\kappa\frac{b(1-\alpha)-1}{1-b\bar{\eta}}\,,\\
\frac{n_0}{n} &= \frac{\mu(1-b\bar{\eta})}{b\mu(1-\alpha-\bar{\eta})+\left(f_0\mathcal{F}_r+\kappa\dfrac{b(1-\alpha)-1}{1-b\bar{\eta}}\right)(1-\bar{\eta}-b\bar{\eta}\alpha)}\,,\\
\frac{n_i}{n_0} &= \alpha_i\frac{f_0\mathcal{F}_r+\kappa\frac{b(1-\alpha)-1}{1-b\bar{\eta}}}{\kappa\left(1+\eta_i\dfrac{b(1-\alpha)-1}{1-b\bar{\eta}}\right)-f_0\mathcal{F}_r(r_i-\eta_i)}\,.
\end{aligned} \tag{30}$$

Just as in the case when the growth rates are all equal (eq. [**9**] in the main text), the distribution of spacers shows a linear dependence on the acquisition probability α_i.

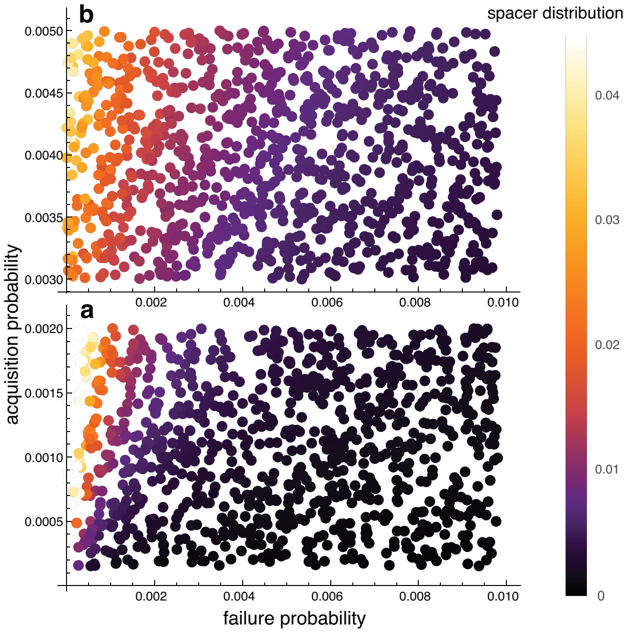

Fig D: Distribution of spacers as a function of failure probability (η_i) and acquisition probability (α_i). The plot was obtained using eqs. (30) for a set of 100 spacers with failure probability below the critical value $\eta < \eta_c$ and acquisition probabilities chosen uniformly in two different regimes. Panel **a**) First regime of small overall acquisition probability $\alpha = \sum \alpha_i \approx 0.1$. As explained in the main text, this leads to a very homogenous population where the best spacers are more abundant. There is a strong dependence on the failure probability, which can be seen by the presence of closely spaced almost vertical contour lines. Panel **b**) Second regime of large overall acquisition probability, $\alpha \approx 0.4$. This tends to reduce the importance of differences in failure probability as shown by contour lines spaced far apart that bend horizontally.

Fig. D shows how the fraction of the bacterial population containing a specific spacer (n_i/n) depends on that spacer's failure probability (η_i) and acquisition probability (α_i). This is shown for the case when all the growth rates are equal ($r_i = 1$). Compare this to Fig. 4 in the main text, which gives a different way of looking at these results.

References

L. M. Childs, N. L. Held, M. J. Young, R. J. Whitaker, and J. S. Weitz, Evolution **66**, 2015 (2012).

W. Jiang, I. Maniv, F. Arain, Y. Wang, B. R. Levin, and L. A. Marraffini, PLoS Genetics **9** (2013), ISSN 15537390.

R. J. Weld, C. Butts, and J. A. Heinemann, Journal of Theoretical Biology **227**, 1 (2004), ISSN 00225193.
